# Reproductive Health and Medication Concerns for Patients With Inflammatory Bowel Disease: Thematic and Quantitative Analysis Using Social Listening

**DOI:** 10.2196/jmir.9870

**Published:** 2018-06-11

**Authors:** Michelle Sophie Keller, Sasan Mosadeghi, Erica R Cohen, James Kwan, Brennan Mason Ross Spiegel

**Affiliations:** ^1^ Department of Health Policy and Management Fielding School of Public Health University of California, Los Angeles Los Angeles, CA United States; ^2^ Division of Informatics Department of Biomedical Sciences Cedars-Sinai Medical Center Los Angeles, CA United States; ^3^ Division of General Internal Medicine Department of Medicine Cedars-Sinai Medical Center Los Angeles, CA United States; ^4^ Department of Medicine University of Arizona Phoenix, AZ United States; ^5^ Division of Gastroenterology Department of Medicine Cedars-Sinai Medical Center Los Angeles, CA United States; ^6^ Cedars-Sinai Center for Outcomes Research and Education Cedars-Sinai Medical Center Los Angeles, CA United States

**Keywords:** pregnancy, breastfeeding, reproductive health, social media, medication adherence, infodemiology, pharmacovigilance

## Abstract

**Background:**

Inflammatory bowel disease (IBD) affects many individuals of reproductive age. Most IBD medications are safe to use during pregnancy and breastfeeding; however, observational studies find that women with IBD have higher rates of voluntary childlessness due to fears about medication use during pregnancy. Understanding why and how individuals with IBD make decisions about medication adherence during important reproductive periods can help clinicians address patient fears about medication use.

**Objective:**

The objective of this study was to gain a more thorough understanding of how individuals taking IBD medications during key reproductive periods make decisions about their medication use.

**Methods:**

We collected posts from 3000 social media sites posted over a 3-year period and analyzed the posts using qualitative descriptive content analysis. The first level of analysis, open coding, identified individual concepts present in the social media posts. We subsequently created a codebook from significant or frequently occurring codes in the data. After creating the codebook, we reviewed the data and coded using our focused codes. We organized the focused codes into larger thematic categories.

**Results:**

We identified 7 main themes in 1818 social media posts. Individuals used social media to (1) seek advice about medication use related to reproductive health (13.92%, 252/1818); (2) express beliefs about the safety of IBD therapies (7.43%, 135/1818); (3) discuss personal experiences with medication use (16.72%, 304/1818); (4) articulate fears and anxieties about the safety of IBD therapies (11.55%, 210/1818); (5) discuss physician-patient relationships (3.14%, 57/1818); (6) address concerns around conception, infertility, and IBD medications (17.38%, 316/1818); and (7) talk about IBD symptoms during and after pregnancy and breastfeeding periods (11.33%, 206/1818).

**Conclusions:**

Beliefs around medication safety play an important role in whether individuals with IBD decide to take medications during pregnancy and breastfeeding. Having a better understanding about why patients stop or refuse to take certain medications during key reproductive periods may allow clinicians to address specific beliefs and attitudes during office visits.

## Introduction

### Inflammatory Bowel Disease and Reproductive Health

Inflammatory bowel disease (IBD) is a chronic, relapsing, and remitting autoimmune disorder comprising Crohn disease (CD) and ulcerative colitis (UC). There are over 1.6 million Americans living with IBD and as many as 70,000 new cases are diagnosed annually [1]. IBD is frequently diagnosed in the second to fourth decades of life, with the highest incidence between 20 and 29 years of age, during peak female reproductive years [2].

Treatment of IBD patients during their reproductive years provides a unique clinical challenge for clinicians, who seek to maintain remission during conception and pregnancy while ensuring the health of the fetus. Adverse outcomes such as preterm delivery, low birth weight, and increased risk of miscarriage are all associated with the degree of disease activity at the time of conception [3,4]. Women who plan their pregnancies while in remission are less likely to experience disease flares during pregnancy and have similar fertility rates as the general population [5]. A growing body of literature now suggests that the majority of IBD medications are low risk for use during pregnancy and even during lactation [6]. Despite this, observational studies note that women—particularly those with CD—have higher rates of voluntary childlessness due to concerns surrounding medication side effects, passing the disease to their offspring, fear of infertility, and advice given by treating physicians [7,8]. Moreover, many women perceive medications to be unsafe during pregnancy [9-11]. Consequently, women are uncertain about taking medications during pregnancy and many believe that medication use should be highly restricted during pregnancy, even when discontinuing medication use might threaten the health of the mother [10]. In one study, one-third of women believed that all medication use should be halted during pregnancy, and 20% of patients surveyed said they would stop medications even if they were advised by a physician to continue medication use [12]. Of the women surveyed, 68% reported anxiety or worries related to the effects of drugs on their pregnancy [12].

### Social Listening and Pharmacovigilance

Social media use is widespread in the United States. More than two thirds of Americans report using Facebook every day, and the typical American is on 3 social media platforms [13]. Additionally, millions of individuals around the world use the internet to describe and share their health care experiences while seeking a sense of community for information-seeking purposes. A study reported that 34% of Americans reported reading about someone else’s health care experience and 15% consulted online reviews of medical facilities [14]. Social listening, or examining social media and forum posts, can reveal trends in public attitudes and behaviors about various health care topics. For example, our group has used social listening to examine patient beliefs about the use of opioid medications and their gastrointestinal side effects, public opinions about the use of virtual reality in health care, and patient beliefs about biologic medications in IBD [15-17]. Researchers have used social listening methods to examine e-cigarette attitudes and posts in the United States, estimate trends in influenza epidemics, and monitor the use and misuse of antidepressants [18-22]. This approach offers unique advantages compared with traditional interviews or focus groups, including the ability to capture discussions among social media users without a moderator present. As a result, social listening may capture opinions from individuals on sensitive topics or from those who might not necessarily choose to participate in a research study [23]. Participants also create communities within social media and network forums that draw patients from many different countries; gathering such a diverse and large group of participants for a focus group or interview-based study would be both logistically challenging and prohibitively expensive.

The percentage of adults who use at least 1 social media site has increased steadily since 2006. Individuals of certain demographic groups are more likely to use and engage in social media compared with other groups. One exception is race—approximately the same proportion of black, white, and Hispanic Americans use at least one social media site [24]. However, income, gender, and education have been shown to be associated with different levels of social media use. A larger proportion of individuals making more than $75,000 per year (77%) reported using at least 1 social media site, compared with 63% of Americans making less than $30,000 per year [24]. Women are also more likely to use social media compared with men [24]. Education also plays an important role—with 79% of Americans with a college degree reporting use of at least 1 social media site compared with 60% of Americans with high school or less education [24]. Age is also an important factor. Although older Americans are increasingly using social media, a greater proportion of younger adults are on social media compared with older adults. A total of 88% of 18- to 29-year-olds in the United States report using some form of social media compared with 37% of Americans 65 years and older [13]. These numbers have some implications for social listening, with a potential bias toward capturing voices from younger individuals with higher incomes and more education, and potentially capturing more female voices.

In this analysis, we examined social media and health forum posts to explore how patients with IBD understand, discuss, and act on the perceived risks and benefits associated with taking IBD medications during conception, pregnancy, and breastfeeding outside of the physicians’ office. The objective of this study was to gain a more thorough understanding of how individuals taking IBD medications during key reproductive periods make decisions about their medications to inform clinical practitioners. Having a better understanding about why patients stop or refuse to take certain medications during key reproductive periods may allow clinicians to address specific beliefs and attitudes during office visits.

## Methods

### Social Media Data Mining

We collaborated with researchers from Treato, a social media data mining service, to find relevant patient experience data. Treato uses Natural Language Processing computer algorithms to collect and index patient, family, and caregiver content from over 3000 websites and social media sites such as Facebook, Healingwell, and TheBump. Posts are indexed using a lexicon of over 100,000 medical terms based on the Unified Medical Language System.

To find IBD-related posts, we extracted relevant data from an e-forum and social media database, using a set of relevant keywords developed through a literature review (Textbox 1). We sought to identify posts about medication use among individuals with IBD and developed 2 category lists—the Conditions category, which included names and abbreviations for IBD, UC, and CD; and the Medications category, which included brand and generic names and abbreviations for medications indicated to treat IBD, including biologic therapies. Posts were selected for analysis if they contained a keyword from the Conditions category as well as a keyword from the Medications category. We further culled posts by using pregnancy and fertility keywords to identify discussions relevant to this study. We analyzed English language posts published online between June 27, 2012, and June 27, 2015. Textbox 1 provides the list of the keywords used.

### Qualitative Content Analysis

After identifying relevant posts, we coded the resulting text corpus using ATLAS.ti (Berlin, Scientific Software Development), a qualitative coding software program. We used qualitative content analysis methods to code the data [25,26]. The coding group was composed of a multidisciplinary team, including MSK, a social science researcher; SM, a board-certified internist; ERC, a board-certified internist and gastroenterology fellow; and JLK, a research intern. The multidisciplinary composition of the team allowed for rich and varied discussions about the codes and the organization of the codes into larger thematic categories. The first level of analysis, open coding, identified individual concepts present in the social media posts. The unit of analysis was the entire post. We coded individual user’s actions and focused on what users were *doing* (eg, sharing personal experiences with medications, expressing fear about medications, not taking medications due to beliefs about harm). We subsequently created a codebook from significant or frequently occurring codes in the data. After creating the codebook, we reviewed the data and coded using our focused codes. Posts were assigned a code if they mentioned experiences regarding pregnancy, breastfeeding, infertility, and experiences with IBD, IBD medications, or IBD-related complications. Each major theme was constructed by aggregating several focused codes identified through the open coding process. For example, focused codes for the major theme “Fear, Anxiety, and Uncertainty” included the following: fear about transferring IBD through genetics, fear of birth defects, fear of delivery issues with IBD, fear of fertility issues with IBD, fear of risks from medications during breastfeeding, fear of risks from medications during pregnancy, fear that pregnancy would worsen IBD symptoms, and general fears and anxieties.

The data collected contained no personal identifiers, and posts included only the website where the comments were posted. Although we were not able to ascertain the gender of the user, given that most social media posts referred to personal experiences with pregnancy, we assumed that most posts were written by women. However, we did find a few exceptions where partners posted on behalf of their wives or where men posted about male fertility issues related to IBD. The social media data were considered part of the public domain. The study was reviewed and approved by the Cedars-Sinai Medical Center IRB. No individual subjects were contacted.

**Keyword searches used to capture social media posts related to reproductive health and inflammatory bowel disease (IBD). The asterisk indicates a search term which was broadened by finding all words that start with the same letters.**

- **Fertility**: fertil*|sperm|embryo|fetus|ttc|infert*|invitro|in vitro|
- **Breastfeeding**: breastfeed*|breast milk|breast*|formula*|breastmilk
- **Pregnancy**: preg*|birth|conception|obgyn|preterm|thebump*|conceive|conception|labor|delivery|trimester|womb|c-section|csection|childbirth|prenatal| morning sickness|twins|babies|newborn*| infant*|placenta*|trisomy|pg
- **Birth defects**: Birth defect|defect*
- **Biologics**: Remicade|humira|cimizia|cimzia
- **Ulcerative colitis**: ulcerative colitis|UC (match case)

## Results

### Principal Results

We identified 1818 unique posts discussing the use of medication in IBD related to fertility, pregnancy, birth outcomes, or breastfeeding. We identified 7 major themes through the open coding process, presented in Table 1. Individuals used social media to (1) seek and share information and advice about medication use during pregnancy, infertility, and breastfeeding, 13.92% (252/1818); (2) express beliefs about the safety of therapies during pregnancy and breastfeeding, 7.43% (135/1818); (3) discuss decisions around personal experiences with medication use, including changes in medications or decisions to stop using medications during pregnancy and breastfeeding, 16.72% (304/1818); (4) articulate fears and anxieties about the safety and use of therapies during pregnancy and breastfeeding, 11.55% (210/1818); (5) discuss physician-patient relationships and related issues of trust and distrust, 3.14% (57/1818) and recommendations from providers about medication use during pregnancy and breastfeeding 3.74% (68/1818); (6) share issues around infertility, including concerns about male infertility and IBD medications, 16.97% (309/1818); and (7) discuss issues around pregnancy and breastfeeding health, including IBD symptoms and medication side effects during pregnancy and post-pregnancy, 11.33% (206/1818).

Our analysis revealed that many factors influence the development of beliefs about IBD medications, including anxieties and fears about taking medications during pregnancy and breastfeeding, the individuals’ trust in their health care providers, beliefs about taking medications associated with infertility, and information gathered from online sources and online communities. Beliefs about IBD medications, in turn, are associated with decisions to start, stop, or change IBD medications during pregnancy, breastfeeding, and infertility treatments.

**Table 1:** Major themes identified in qualitative analysis of social media regarding reproductive health and Inflammatory Bowel Disease (IBD).

Themes^a^		n^b^ (%)
**Expressing beliefs about safety**		135 (7.43)
	Belief that benefits of IBD medications outweigh risks	32 (1.76)
	Belief that IBD medication is not safe	35 (1.93)
	Belief that IBD medication is safe	68 (3.74)
**Sharing decisions about taking, changing, or stopping IBD medications**		304 (16.72)
	Changed, changing, or will change medication during pregnancy or while breast feeding	13 (0.72)
	Took, is taking, or will take medication during pregnancy or while breast feeding	198 (10.89)
	Stopped, stopping, or will stop medication during pregnancy or while breastfeeding	93 (5.12)
**Expressing fear, anxiety, and uncertainty**		210 (11.55)
	Fear and anxiety surrounding medication and pregnancy and or breastfeeding	156 (8.58)
	Uncertainty about use of IBD medication during pregnancy and or breastfeeding	54 (2.97)
**Receiving recommendations from providers**		68 (3.74)
	Provider advised that IBD medications are safe	35 (1.76)
	Provider advised that IBD medications are unsafe	23 (1.27)
	Provider unsure about safety of IBD medications	10 (0.55)
**Discussing patient-physician relationships**		57 (3.14)
	Poor communication and or evidence of distrust between patient and provider	38 (2.09)
	Good communication or user expresses trust in provider	19 (1.05)
**Giving and seeking advice experiences**		252 (13.86)
	Seeking advice about IBD and reproductive health	156 (8.58)
	Giving advice about IBD and reproductive health	97 (5.34)
**Reporting health during pregnancy**		202 (11.11)
	Reported healthy pregnancy	65 (3.58)
	Reported flare-ups during pregnancy	65 (3.58)
	Reported flare-ups postpartum	64 (3.58)
	Onset of IBD coincided with pregnancy	37 (2.04)
**Discussing infertility**		309 (16.97)
	General infertility issues	292 (16.06)
	Male infertility	19 (1.04)

^a^Subtheme totals may not add up to main theme totals due the co-occurrence of codes. Percentages are calculated using the 1818 total coded posts.

^b^The unit of analysis was the social media post. The data did not include personal identifiers, so it is possible that the same individual posted multiple times on different websites.

### Giving and Seeking Advice Around Inflammatory Bowel Disease and Medications

We identified 252 posts (13.92%) where individuals sought and shared advice with others on taking various types of medications, treating flare-ups, and addressing labor complications during pregnancy and breastfeeding. Many individuals expressed distress about recommendations from physicians or about information they had gathered online, particularly with regards to medication changes. For example, a woman 20 weeks into her pregnancy wrote about continuing Remicade infusions (a biologic treatment for IBD) until the 32-week mark and then discontinuing infusions and starting prednisone. She wrote:

They don’t know the full effects of Remicade and breast milk so I will not be having anymore infusions after my infusion in September...My Perinatalogist said most likely the best option to control it will be Prednisone...Is Prednisone really my only/the best option until I’m done nursing?

The woman in the post above expressed uncertainty in both the medical and the scientific community and the clinicians’ recommendations. Hesitation in trusting clinical findings and the medical community as a whole recurred throughout the posts in our analysis.

Individuals also expressed anguish about the lack of definitive safety information available for newer medications, particularly biologics, and sought advice on these medications. For instance, 1 individual seeking advice about his/her partner’s treatment wrote:

Any advice or questions we should ask our doctor would be great. Humira is so new that most Dr’s don’t have a clue when we ask about complications.

The individual noted that his or her partner had received conflicting information from various providers on medications and delivering via Caesarian section and shared the frustrations with the online community.

### Beliefs About Safety of Inflammatory Bowel Disease Medications

Individuals also expressed their implicit and explicit beliefs about taking IBD medications during pregnancy, breastfeeding, and infertility treatments. Despite many concerns shared about the safety of IBD mediations, there were twice as many posts expressing beliefs that IBD medications are safe during pregnancy and breastfeeding (n=68, 3.74%) as compared with beliefs that IBD medications are not safe (n=35, 1.93%). When sharing their experiences, many individuals explicitly noted that their medications were “very safe” and mentioned whether their providers agreed with their beliefs. One individual emphasized that she had consulted with 3 different clinicians when considering the safety of the IBD medications she was taking while pregnant, sharing both her personal opinion and viewpoints of clinicians in her life:

I personally felt it was quite safe to breastfeed while on mesalamine, as did my OBGYN, GI and sister [she’s an MD].

Other social media users expressed doubts or firm beliefs that IBD medications were unsafe during pregnancy, echoing other qualitative work that has found that many women are uncertain about taking any medications during pregnancy.

Some individuals expressed beliefs that conflicted with recommendations they had received from their providers. Many of these patients expressed distrust in the evidence supporting the safety of IBD medications. For example, 1 patient posted a comment about a bad experience she had heard of regarding an individual using a biologic medication during pregnancy:

Humira has not been fully studied in pregnant women. There is even a registry to have to go on if you plan on being pregnant while you take humira. I know of a horror story and pregnancy and humira [I am not sure it was the humira that caused it or just bad luck.].

We also identified 32 (1.76%) posts where individuals weighed the benefits and risks of taking IBD medication and concluded that the benefits outweighed the risks of a severe flare-up during pregnancy. Avoiding flare-ups by taking IBD medications was a common narrative. One 29-year-old patient with CD discussed taking azathioprine for 8 years and staying on it throughout her pregnancy. She wrote:

If you come off aza before getting preg it is more dangerous for the baby in case you relapse whilst carrying the child and get really poorly! It’s safer for yourself to be [healthy] and well whilst pregnant. I have heard about the birth defect thing (not through professionals though) but it was highly advised that I stay on aza.

She notes the various sources of information she consulted throughout her pregnancy, including information from social networks and formal sources (ie, professionals), highlighting the fact that many individuals seek multiple sources of information about medical decisions and must weigh the validity of these sources when making medication-related decisions.

### Sharing Decisions About Taking, Changing, or Stopping Inflammatory Bowel Disease Medications During Pregnancy and Breastfeeding

Many individuals reported changing or stopping medications during pregnancy and breastfeeding, either as a result of the advice of a clinician or based on personal beliefs about the safety of taking IBD medications. There were 198 posts (10.89%) where individuals specifically cited taking or planning to take IBD medications during pregnancy or breastfeeding, 93 (5.12%) posts where individuals said they stopped or planned to stop medications during pregnancy or breastfeeding, and 13 (0.72%) posts where individuals said they planned on changing or changed medications during these reproductive periods.

Individuals cited reasons such as beliefs about the safety of medications, advice from providers, or advice from fellow sufferers for their decisions. As an example, one social media user discussed her decision to avoid breastfeeding while taking Remicade, a biologic therapy for IBD:

I do not plan on [breastfeeding] while being on remicade. We will formula feed. But I know A LOT of people that do bf, I’m just not comfortable with it.

This individual expressed that although she understood that other people had made the decision to breastfeed while taking these medications, she preferred to take the personal risk of going off the medication to avoid any perceived risk to her baby. This example highlights a tension that many individuals expressed between caring for their own bodies and avoiding perceived harm for the baby. The desire to avoid medications during pregnancy extended to individuals who had already given birth and taken medications while pregnant but wanted to avoid all medications for the next pregnancy. In one post, a woman expressed how she took 2 different IBD medications—Azulfidine and Asacol—during her first pregnancy, which she mentions was unplanned. However, for future planned pregnancies, she describes that she would:

...go off my meds for that 9 months. I would take all types of prenatals and omega and iron, and I would hopefully stay healthy for that baby. That would be MY choice for MY baby.

The individual posting here emphasizes her desire to use nonmedication alternatives (supplements), perceived as safer, to stay “healthy” and thus potentially be able to avoid taking medications she perceives as harmful.

Among those who cited either a personal reason or provider advice to stop taking a medication, nearly 3 times as many patients who stopped taking a medication did so as a result of personal reasons (N=25, 1.38%) versus those who stated they stopped based on the advice of a provider (N=8, 0.44%). One post exemplifies this decision-making process:

I am supposed to take Asacol HD. However I haven’t taken it with my pregnancy for my own personal reasons especially since I do feel pretty good.

### Expressing Fear, Anxiety, and Uncertainty

Patients frequently expressed fear, anxiety, and uncertainty around the use of IBD medications during periods of infertility, pregnancy, and breastfeeding. A major concern was the fear of birth defects and higher risk of miscarriage due to taking any medications during pregnancy. Although many patients had received medical advice, they frequently looked to others online to validate their concerns and gather more information. For instance, an individual with UC who was 7 months pregnant wrote:

My doctor now wants me to take Asacol HD and I’m very hesitant to take any medication while pregnant for fear that it may cause some kind of issue or birth defect with my baby.

Patients were conflicted between their fears of potential harms to the baby and the benefits of taking medications during pregnancy and breastfeeding, despite reassurances from clinicians. In many cases, providers appeared to express uncertainty with regards to the safety of the medication, leading the patient to also feel a heightened degree of uncertainty. One individual wrote:

My doc said I could do Rowasa and he thought I could still nurse “with caution” and watch the baby's weight gain and BM’s, or I would need to cease nursing and take Canasa and Lialda. I hate taking pills. I only nurse my son once per day now because I am afraid of the effects of the Rowasa on him, but hate to give up that one session.

### Discussing Patient-Physician Relationships and Medication Choices

Patients discussed beliefs that their physicians expressed about the safety and appropriate use of IBD medications during pregnancy and breastfeeding. There were 35 reports (1.93%) of providers stating that IBD medications were safe during pregnancy/breastfeeding, 23 (1.27%) where patients said the provider indicated that IBD medications were unsafe during these periods, and 10 (0.55%) where patients said their provider was unsure about the safety of IBD medications during pregnancy and breastfeeding.

In this example, an individual discusses her experiences with her maternal-fetal medicine specialist, the beliefs her provider has expressed, and the relationship she has with this provider:

...My maternal-fetal medicine doctor says Humira is perfectly safe for pregnancy and breastfeeding (class B drug)...I really recommend seeing an MFM doctor if you haven’t already, even just for consultation. Mine knew a LOT about this stuff. More than my OBGYN, and even more than my IBD specialist. It was a very reassuring experience and has allowed me to enjoy my pregnancy!

In the post above, the individual expressed how the maternal-fetal medicine doctor was the one who was able to bridge the information divide between the 2 other specialists.

This experience echoes that of many individuals who posted on social media, many of whom expressed frustration that their obstetricians were not well informed on how to counsel women with IBD and how their gastroenterologists did not provide sufficient counseling during pregnancy. Many patients wrote that they were frustrated with their providers’ unwillingness to discuss pregnancy and fertility issues. One patient expressed anguish regarding her conversations with physicians about IBD medications and pregnancy:

I’m so confused. None of my docs even want to talk about pregnancy! They say, well you really want to discuss this when you’re on all these meds? Hmm, yes! Docs can be so frustrating at times. Possibly should I see an OBGYN?

We found that even when patients discussed provider recommendations, there were elements of distrust or uncertainty regarding the physicians’ advice. One individual noted:

My doctors were scaring me saying it was bad but I’ve been reading online that a lot of women actually stay on it.

### Expressing Concerns About Infertility and Inflammatory Bowel Disease Medications

We identified a group of posts focused on the effects of IBD and IBD medications on infertility (n=316, 17.38%). In these posts, social media users also discussed the effect of IBD or IBD medications on infertility in general or whether these affected the effectiveness of in vitro fertilization. Although most posts were related to female infertility and IBD, we identified 19 posts (1.05%) discussing male infertility or risks of birth defects due to medications taken by male IBD patients. Several patients reported confusion or surprise that medications used to manage IBD could affect male fertility. One patient with CD taking Cimzia and prednisone with a persistent flare-up sought advice on taking methotrexate while he and his wife attempted to conceive:

...I have never done anything like this before but my wife and I need help....My wife and I are most worried about having children soon or in the future but based on my research, you should not try while on the medication,. Does anyone know any info on this? Please help!

As with other posts, the individual decided to turn to social media to seek information rather than turn to a medical professional.

### Communicating About General Pregnancy Health and Inflammatory Bowel Disease

Patients used social media to discuss IBD symptoms and potential causes during pregnancy. We found 202 posts (11.11%) that discussed flare-ups during and after pregnancy/during breastfeeding. Patients discussed how changes during pregnancy and breastfeeding affected their IBD symptoms; 65 posts (3.57%) discussed an IBD flare-up during pregnancy, 65 posts (3.57%) discussed complete remission during pregnancy, and 64 posts reported postpartum flare-ups. We also found 37 posts (2.04%) where patients reported that the onset of IBD coincided with their pregnancy, underscoring the importance of patient education on IBD and reproductive issues during the reproductive years.

## Discussion

### Principal Findings

We found that social media users often expressed significant tension between taking IBD medications for their own health and fears about potential birth defects. Our findings are in line with another research that finds that individuals often overestimate the teratogenic effects of medications during pregnancy [10]. A study using traditional survey methods also found that one quarter of participants thought it was more important to tolerate flares than take medications during pregnancy and 36% expressed beliefs that IBD medications are harmful to the fetus [27]. Although we were not able to measure education in our study, other studies have found that education level and health literacy are inversely associated with beliefs about medication safety during pregnancy, with less educated women/women with lower health literacy stating beliefs that medications are harmful during pregnancy [10,28].

Additionally, other studies have also found that adherence to medications for chronic conditions is low during pregnancy and is strongly related to women’s beliefs about medication safety [29]. Our findings demonstrate that social media users indeed express that they stop taking medications for IBD during pregnancy when they are feeling better to reduce perceived risks.

Discussions with gastroenterologists about reproductive health in IBD are vital to bridge information gaps. Women with IBD are more likely to seek information about contraception and reproductive health from their gastroenterologist than from a family care physician, although most of this counseling is initiated by patients [29]. Yet, most patients seeing a gastroenterologist have no documented reproductive counseling, and 41% of general practitioners report that they never raise the issue of family planning with their IBD patients [12]. A timely discussion with a provider can alleviate some of these anxieties and help women make better decisions about managing their IBD during pregnancy. Discussion of family planning with a physician is associated with higher IBD-related pregnancy knowledge scores and lower odds of voluntary childlessness [8]. Gastroenterologists can play an important role in transmitting information about reproductive health and IBD during office visits to increase adherence to medications and reduce flare-ups.

One potential issue raised by social media users in this study may be the dearth of evidence-based information about medication safety during pregnancy, particularly for newer medications such as biologics. A 2010 study of 305 obstetricians and gynecologists found that 42% selected lack of sufficient information on medications as a significant barrier to discussing medication safety with pregnant women [30]. Increasing pregnancy registry participation could improve the quality and quantity of information available to clinicians and patients.

### Strengths and Limitations

Strengths of this research include the ability to analyze the varied perspectives of hundreds of patients from all over the globe, which would be logistically unfeasible with traditional focus groups or interviews. However, there are also several limitations to this analysis. First, we cannot verify the identities of the patients posting on social media, particularly because many post using screen names. As a result, we cannot definitely confirm whether all patients have clinically verified diagnoses or are self-diagnosed. Another limitation is that not all patients are equally likely to write about their medical experiences online. As a result, we may only have the opportunity to view and analyze the experiences of a certain segment of the population.

As other researchers have found, social media use is associated with female gender, higher income, higher level of education, and younger age [13,24]. Moreover, these same factors are associated with characteristics of individuals who seek health information online [31]. Health status may be associated with online information-seeking, but the findings are conflicting—one study identified that healthier individuals are more likely to seek information online, whereas another study found that health status was not associated with seeking online information [31,32]. Thus, it is possible that the social media posts included in this analysis reflect a younger, more educated, and higher-income cohort, and thus, the results may be more applicable to individuals with similar backgrounds. As with traditional focus groups, another limitation is that certain individuals may be more likely to engage and participate in the conversations [33]. However, we examined more than 1800 posts from a variety of different websites, which should allow for discussions and posts from a variety of different communities and individuals, which strengthens the findings from our analyses.

### Conclusions

In this study, we used social listening methods to survey how individuals with IBD think about and make decisions about medications during key reproductive periods. We aimed to understand the types of beliefs that individuals have about IBD medications and birth defects, fertility, or outcomes in children. One important finding of our study is that individuals’ needs may not be adequately addressed in clinical practice and that many patients are frustrated with their providers for failing to discuss the effects of their pharmacological regimens on fertility, pregnancy, and breastfeeding. We found that both men and women have concerns about the safety and effects of IBD therapies during reproductive periods, and that there are significant fears and anxieties about the use of these therapies that are largely not addressed during office visits. As result, individuals may turn to online groups for information and emotional support. We also found that individuals often questioned the recommendations of their clinicians and sought second opinions via online forums. However, as these online forums may not provide evidence-based or updated medical information, individuals might receive erroneous or dangerous information. Future research should examine why clinicians might be hesitant to bring up reproductive concerns during office visits for individuals with IBD. Future research might also elucidate successful strategies that individuals can use to raise these topics that foster effective shared decision making in the IBD and reproductive health settings. Improving patient-provider communication around this issue may help improve the health of individuals with IBD during important reproductive periods.

## Abbreviations

IBD: inflammatory bowel disease
CD: Crohn disease
UC: ulcerative colitis
